# Crystal structure of a 1:1 adduct of tri­phenyl­tin chloride with 3-cyclo­hexhyl-2-phenyl-1,3-thia­zolidin-4-one

**DOI:** 10.1107/S2056989019001592

**Published:** 2019-02-08

**Authors:** Hemant P. Yennawar, John Tierney, Kevin C. Cannon

**Affiliations:** a The Pennsylvania State University, Department of Biochemistry and Molecular Biology, University Park, PA 16802, USA; b Pennsylvania State University, Brandywine Campus, Department of Chemistry, Brandywine, PA 19063, USA; c The Pennsylvania State University, Department of Chemistry, Abington College, Abington, PA 19001, USA

**Keywords:** crystal structure, thia­zolidin-4-one, tin complex, C—H⋯Cl-metal hydrogen bond

## Abstract

In the centrosymmetric (racemic) title compound, chlorido­(3-cyclo­hexhyl-2-phenyl-1,3-thia­zolidin-4-one-κ*O*)tri­phenyl­tin(IV), [Sn(C_6_H_5_)_3_Cl(C_15_H_19_NOS)], the tin(IV) atom exhibits a trigonal–bipyramidal coordination geometry with the three phenyl groups in equatorial positions and the chloride anion and ligand oxygen atom present at axial sites [O—Sn—Cl = 175.07 (14)°]. The thia­zolidinone ring of the ligand adopts an envelope conformation with the S atom as the flap. The dihedral angles between the heterocycle ring plane (all atoms) are 44.3 (9)° with respect to the pendant *C*-phenyl plane and 34.3 (11)° to the *N*-cyclo­hexyl ring (all atoms). The *C*-phenyl and *N*-cyclo­hexyl ring are close to orthogonal to each other, with a dihedral angle of 81.1 (4)° between them. In the crystal, mol­ecules are linked by weak C—H⋯Cl hydrogen bonds to generate [001] chains.

## Abstract 2

This is the second reported crystal structure of an adduct of a 2,3-disubstituted 1,3-thia­zolidine-4-one ligand and tri­phenyl­tin chloride. The tin atom adopts a trigonal–bipyramidal coordination geometry with the O and Cl atoms in the axial sites.

## Chemical context   

Substituted 1,3-thia­zolidin-4-ones themselves as well as ligands attached to various metals exhibit a wide range of biological activity (Jain *et al.*, 2012 ▸; Kozlowski *et al.* 2002 ▸). The ligand of the title compound, (*N*)-3-xyclohexyl-2-phenyl-1,3-thia­zolidine-4-one, is easily prepared from *N*-cyclco­hexyl­idene aniline and thio­glycolic acid utilizing a method originally proposed by Surrey (1947 ▸). The crystal structure of (*N*)-3-cyclo­hexyl-2-phenyl-1,3-thia­zolidine-4-one has previously been reported (Cannon *et al.* 2013 ▸), as have a number of other 2,3-disubstituted-thia­zolidin-4-one structures (Yennawar *et al.*, 2017 ▸; Vigorita *et al.*, 1979 ▸). Furthermore, the X-ray crystal structure of 2,3-diphenyl-1,3-thia­zolidin-4-one as a 1:1 adduct with tri­phenyl­tin chloride has been described **(**Smith *et al.* 1995 ▸ **)**, and along with related complexes has biological activity against *Cerotysistis Ulmi*, the fungus that causes Dutch Elm Disease (Beraldo & de Lima, 2008 ▸).
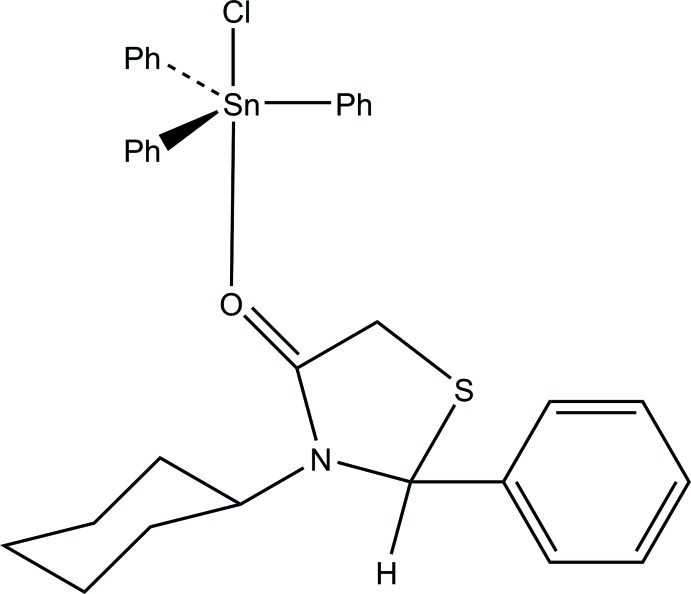


Herein, we report the synthesis and crystal structure of the 1:1 adduct of tri­phenyl­tin chloride with (*N*)-3-cyclo­hexhyl-2-phenyl-1,3-thia­zolidin-4-one.

## Structural commentary   

**Figure 1:**
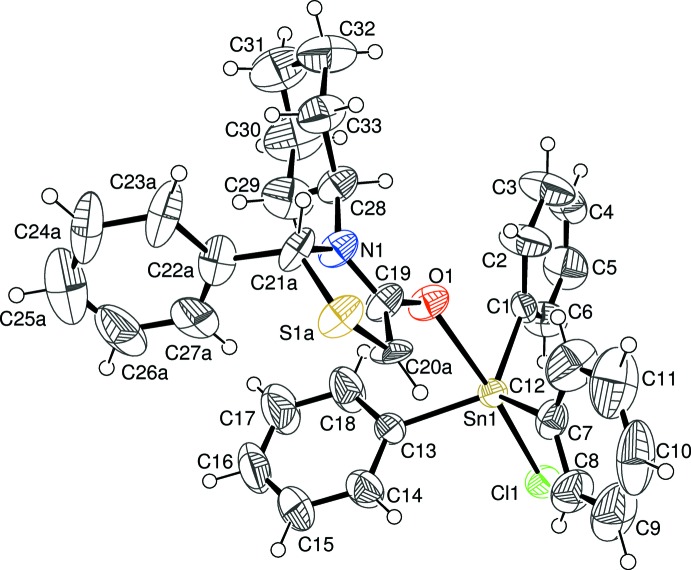
The mol­ecular structure of the title compound with displacement ellipsoids drawn at the 40% probability level. Only one disorder component of the thia­zolidinone ring and its attached C22 phenyl ring are shown.

The title compound (Fig. 1 ▸) shows a five-coordinate geometry around the tin atom (Table 1 ▸) with three phenyl groups placed equatorially, and a chloride ligand and an O-bonded thia­zolidinone ligand at the axial sites. The Cl—Sn—O(ligand) principal axis is almost 5° off its ideal linear geometry with a bond angle of 175.07 (14)°. The (*N*)-3-cyclo­hexhyl-2-phenyl-1,3-thia­zolidin-4-one ligand contains a chiral center at the 2-carbon atom (C21): in the arbitrarily chosen asymmetric unit, this atom has an *R* configuration, but crystal symmetry generates a racemic mixture.

The most closely related structure previously reported is that of 2,3-diphenyl-1,3-thia­zolidin-4-one as a 1:1 adduct with tri­phenyl­tin chloride (Smith *et al.*, 1995 ▸). Since this mol­ecule had a less bulky phenyl group at N3 (N1 in our numbering scheme) than the more bulky cyclo­hexyl group, the principal angle is almost exactly linear at 179.2°. Previously, using Mössbauer effect spectroscopy, the 2,3-diphenyl-1,3-thia­zolidin-4-one as a 1:1 adduct with tri­phenyl­tin chloride gave an *r* value (the ratio of quadrupole splitting to isomer shift) of 2.41, indicative of the tin with a coordination number greater than four. Although Mössbauer spectroscopy was not used in our study, we see the same coordination properties with the title mol­ecule in the X-ray structure. The Sn—O bond length was found to be 2.500 Å for the tin–di­phenyl­thia­zolidinone adduct, using Mössbauer techniques as well as the X-ray data, whereas, the X-ray data for the title compound yields an Sn—O bond length of 2.488 (4) Å. These values are almost the same and show no difference in having the presence of phenyl and a cyclo­hexyl group at C2 and N3 (C21 and N1 in our numbering scheme) *versus* a phenyl group at each location.

## Supra­molecular features   

**Figure 2:**
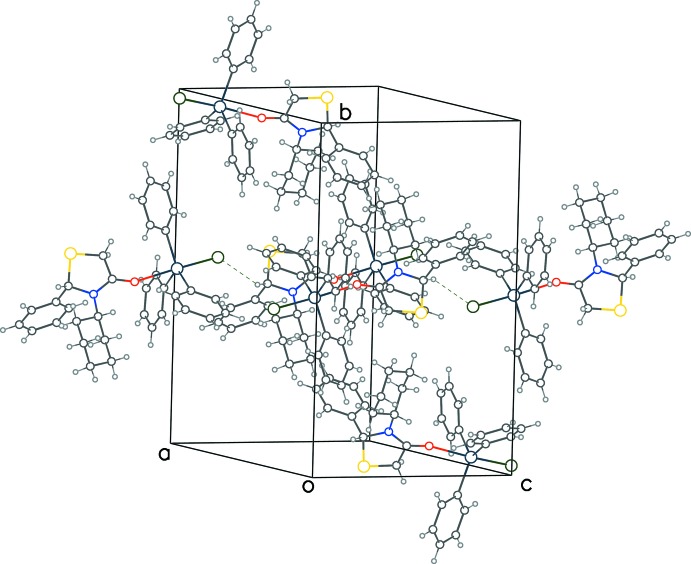
Packing diagram for the title compound with C—H⋯Cl inter­actions indicated by dashed lines.

The surface of the title compound is primarily hydro­phobic due to four aromatic and one aliphatic ring resulting in inter­molecular van der Waals inter­actions (Fig. 2 ▸) between the various aromatic rings. A sole weak hydrogen bond between the chiral carbon atom (C21) with a chloride ion of the neighboring mol­ecule related by translation symmetry in the *c*-axis direction [H⋯Cl = 2.76 Å, C⋯Cl = 3.569 (9) Å, C—H⋯Cl = 140°] helps to consolidate the packing.

## Database survey   

There is only one closely related structure previously reported and that is 2,3-diphenyl-1,3-thia­zolidin-4-one as a 1:1 adduct with tri­phenyl­tin chloride (Smith *et al.*, 1995 ▸).

## Synthesis and crystallization   

The synthesis of (*N*)-3-cyclo­hexyl-2-phenyl-1,3-thia­zolidine-4-one has been previously reported (Cannon *et al.*, 2013 ▸).

The 1:1 adduct with tri­phenyl­tin chloride was prepared by dissolving 0.0023 mol of *N*-3-cyclo­hexhyl-2-phenyl-1,3-thia­zolidin-4-one in 15 ml of acetone and adding this solution dropwise to a 15 mL solution of tri­phenyl­tin chloride (0.0023 mol) in a 50 ml round-bottom flask while stirring at room temperature for 3 h. Stirring was then stopped and the solution was allowed to stand for an additional 10 h. A precipitate was apparent, which was filtered and the filtrate was reduced under vacuum on a rotary evaporator, dried under vacuum to give an oily residue, which formed crystals when heated in ligroin. Recrystallization from ligroin solution yielded 0.0022 mol (97% yield) of the title 1:1 complex in the form of colorless blocks: m.p. 372–375 K (no literature reports).

**Tri­phenyl­tinchloride-3-cyclo­hexyl-2-phenyl-1,3-thia­zolidin-4-one:** Yield (97%); m.p. 372–375 K, cm^−1^ 1658.6 (C=O); ^1^H NMR (CDCl_3_): 7.78–7.27 (20 H, *m*, aromatics), 5.66 (1H, *d*, *J* = 1.9 Hz, C2), 3.89 (1H, *dd*, *J* = 1.9 Hz and *J* = 15.6 Hz, C5), 3.85–3.78 (1H, *m*, NCH), 3.58 (1H, *d*, *J* = 15.6 Hz, C5), 1.79–0.91 (10H, *m*, cyclo­hexyls); ^13^C NMR: 171.77 (C4), 142.98, 137.78, 136.34 (*t*, 25.3 Hz), 130.62, 129.32 (*t*, *J* = 32.2 Hz), 129.07, 128.88, 128.52, 126.38, 62.83 (C2), 56.30, 33.23 (C5), 31.03, 30.12, 26.10, 25.42. C_33_H_34_OClSnNS.

## Refinement   

In spite of our search for a better crystal we had to work with one that was not optimal, as is evident from the high value of *R*_int_ = 0.0721. Upon refinement we observed positional disorder in almost a fourth of the structure (nine out of thirty-eight non-H atoms). As a result, some refinement parameters such as the ADP max/min ratio (8.2) for one of the atoms are slightly above optimal values but the atomic connectivity is clearly established. Crystal data, data collection and structure refinement details are summarized in Table 2 ▸. The H atoms were placed geometrically and allowed to ride on their parent C atoms during refinement, with C—H distances of 0.93 Å (aromatic) and 0.97 Å (methyl­ene), with *U*_iso_(H) = 1.2*U*_eq_ (aromatic or methyl­ene C) or 1.5*U*_eq_(methyl C).

## Supplementary Material

Crystal structure: contains datablock(s) I. DOI: 10.1107/S2056989019001592/hb7780sup1.cif

Structure factors: contains datablock(s) I. DOI: 10.1107/S2056989019001592/hb7780Isup2.hkl

Supporting information file. DOI: 10.1107/S2056989019001592/hb7780Isup3.mol

CCDC reference: 1894217

Additional supporting information:  crystallographic information; 3D view; checkCIF report

## Figures and Tables

**Table 1:** Selected bond lengths (Å)

Sn1—C1	2.141 (4)	Sn1—Cl1	2.4439 (19)
Sn1—C7	2.130 (4)	Sn1—O1	2.488 (4)
Sn1—C13	2.119 (4)		

**Table 2:** Experimental details

Crystal data	
Chemical formula	[Sn(C_6_H_5_)_3_Cl(C_15_H_19_NOS)]
*M*_r_	646.81
Crystal system, space group	Monoclinic, *P*2_1_/*c*
Temperature (K)	218
*a*, *b*, *c* (Å)	15.360 (5), 18.879 (6), 10.992 (3)
β (°)	102.524 (5)
*V* (Å^3^)	3111.8 (17)
*Z*	4
Radiation type	Mo *K*α
μ (mm^−1^)	1.00
Crystal size (mm)	0.15 × 0.11 × 0.10

Data collection	
Diffractometer	Bruker CCD area detector
Absorption correction	Multi-scan (*SADABS*, Bruker, 2001 ▸)
*T*_min_, *T*_max_	0.865, 0.907
No. of measured, independent and observed [*I* > 2σ(*I*)] reflections	24296, 7791, 5009
*R*_int_	0.072
(sin θ/λ)_max_ (Å^−1^)	0.673

Refinement	
*R*[*F* ^2^ > 2σ(*F* ^2^)], *wR*(*F* ^2^), *S*	0.083, 0.221, 1.04
No. of reflections	7791
No. of parameters	365
No. of restraints	133
H-atom treatment	H-atom parameters constrained
Δρ_max_, Δρ_min_ (e Å^−3^)	2.50, −1.17

Computer programs: *SMART* and *SAINT* (Bruker, 2001 ▸), *SHELXS97* and *SHELXL97* (Sheldrick, 2008 ▸) and *OLEX2* (Dolomanov *et al.*, 2009 ▸).

## supplementary crystallographic information

### Crystal data

**Table:**

[Sn(C_6_H_5_)_3_Cl(C_15_H_19_NOS)]	*F*(000) = 1320
*M**_r_* = 646.81	*D*_x_ = 1.381 Mg m^−^^3^
Monoclinic, *P*2_1_/*c*	Mo *K*α radiation, λ = 0.71073 Å
*a* = 15.360 (5) Å	Cell parameters from 4375 reflections
*b* = 18.879 (6) Å	θ = 2.3–26.4°
*c* = 10.992 (3) Å	µ = 1.00 mm^−^^1^
β = 102.524 (5)°	*T* = 218 K
*V* = 3111.8 (17) Å^3^	Block, colorless
*Z* = 4	0.15 × 0.11 × 0.10 mm

### Data collection

**Table:**

Bruker CCD area detector diffractometer	7791 independent reflections
Radiation source: fine-focus sealed tube	5009 reflections with *I* > 2σ(*I*)
Parallel-graphite monochromator	*R*_int_ = 0.072
phi and ω scans	θ_max_ = 28.6°, θ_min_ = 1.7°
Absorption correction: multi-scan (SADABS, Bruker, 2001)	*h* = −16→20
*T*_min_ = 0.865, *T*_max_ = 0.907	*k* = −25→25
24296 measured reflections	*l* = −14→14

### Refinement

**Table:**

Refinement on *F*^2^	Primary atom site location: structure-invariant direct methods
Least-squares matrix: full	Secondary atom site location: difference Fourier map
*R*[*F*^2^ > 2σ(*F*^2^)] = 0.083	Hydrogen site location: inferred from neighbouring sites
*wR*(*F*^2^) = 0.221	H-atom parameters constrained
*S* = 1.04	*w* = 1/[σ^2^(*F*_o_^2^) + (0.0926*P*)^2^ + 6.5369*P*] where *P* = (*F*_o_^2^ + 2*F*_c_^2^)/3
7791 reflections	(Δ/σ)_max_ < 0.001
365 parameters	Δρ_max_ = 2.50 e Å^−^^3^
133 restraints	Δρ_min_ = −1.16 e Å^−^^3^

### Special details

**Table:**

Geometry. All esds (except the esd in the dihedral angle between two l.s. planes) are estimated using the full covariance matrix. The cell esds are taken into account individually in the estimation of esds in distances, angles and torsion angles; correlations between esds in cell parameters are only used when they are defined by crystal symmetry. An approximate (isotropic) treatment of cell esds is used for estimating esds involving l.s. planes.
Refinement. Refinement of F^2^ against ALL reflections. The weighted R-factor wR and goodness of fit S are based on F^2^, conventional R-factors R are based on F, with F set to zero for negative F^2^. The threshold expression of F^2^ > 2sigma(F^2^) is used only for calculating R-factors(gt) etc. and is not relevant to the choice of reflections for refinement. R-factors based on F^2^ are statistically about twice as large as those based on F, and R- factors based on ALL data will be even larger.

### Fractional atomic coordinates and isotropic or equivalent isotropic displacement parameters (Å^2^)

**Table:**

	*x*	*y*	*z*	*U*_iso_*/*U*_eq_	Occ. (<1)
C1	0.5942 (3)	0.0290 (3)	0.2921 (4)	0.0467 (14)	
C2	0.5376 (4)	0.0374 (4)	0.1758 (4)	0.092 (3)	
H2	0.5556	0.0224	0.1045	0.111*	
C3	0.4542 (4)	0.0681 (4)	0.1662 (5)	0.116 (4)	
H3	0.4163	0.0738	0.0884	0.139*	
C4	0.4274 (3)	0.0905 (4)	0.2728 (7)	0.089 (3)	
H4	0.3715	0.1111	0.2664	0.106*	
C5	0.4839 (4)	0.0821 (4)	0.3891 (5)	0.097 (3)	
H5	0.4660	0.0971	0.4604	0.116*	
C6	0.5674 (4)	0.0513 (3)	0.3987 (4)	0.077 (2)	
H6	0.6052	0.0457	0.4765	0.092*	
C7	0.7266 (4)	−0.1279 (2)	0.2527 (5)	0.0585 (17)	
C12	0.6759 (5)	−0.1492 (3)	0.1380 (5)	0.108 (4)	
H12	0.6395	−0.1167	0.0872	0.130*	
C11	0.6795 (5)	−0.2190 (4)	0.0993 (6)	0.132 (4)	
H11	0.6455	−0.2333	0.0225	0.158*	
C10	0.7338 (6)	−0.2676 (2)	0.1753 (8)	0.126 (4)	
H10	0.7362	−0.3143	0.1494	0.151*	
C9	0.7845 (5)	−0.2463 (3)	0.2900 (8)	0.123 (4)	
H9	0.8209	−0.2788	0.3409	0.148*	
C8	0.7809 (4)	−0.1765 (3)	0.3287 (5)	0.085 (3)	
H8	0.8148	−0.1622	0.4055	0.102*	
C13	0.8355 (3)	0.0451 (2)	0.3297 (6)	0.0504 (14)	
C18	0.8226 (3)	0.1176 (3)	0.3135 (7)	0.104 (4)	
H18	0.7653	0.1364	0.2996	0.125*	
C17	0.8953 (5)	0.1621 (2)	0.3179 (9)	0.141 (6)	
H17	0.8867	0.2107	0.3071	0.169*	
C16	0.9809 (4)	0.1341 (3)	0.3387 (8)	0.115 (4)	
H16	1.0296	0.1639	0.3417	0.138*	
C15	0.9938 (3)	0.0615 (4)	0.3550 (7)	0.092 (3)	
H15	1.0511	0.0428	0.3688	0.111*	
C14	0.9211 (3)	0.0170 (2)	0.3505 (6)	0.075 (2)	
H14	0.9298	−0.0315	0.3614	0.090*	
C19	0.7557 (6)	0.0101 (4)	0.0116 (7)	0.0592 (19)	
C20B	0.839 (2)	−0.0392 (13)	0.0291 (17)	0.068 (6)	0.66 (6)
H20A	0.8263	−0.0855	0.0595	0.081*	0.66 (6)
H20B	0.8894	−0.0187	0.0872	0.081*	0.66 (6)
C21B	0.810 (2)	0.0427 (14)	−0.166 (3)	0.064 (5)	0.66 (6)
H21B	0.7775	0.0400	−0.2531	0.077*	0.66 (6)
C22A	0.864 (3)	0.0842 (15)	−0.190 (4)	0.065 (9)	0.34 (6)
C23A	0.872 (4)	0.1077 (19)	−0.307 (4)	0.098 (15)	0.34 (6)
H23A	0.8325	0.0919	−0.3783	0.117*	0.34 (6)
C24A	0.940 (5)	0.155 (2)	−0.317 (5)	0.12 (2)	0.34 (6)
H24A	0.9452	0.1706	−0.3956	0.147*	0.34 (6)
C25A	0.999 (4)	0.179 (2)	−0.211 (6)	0.13 (2)	0.34 (6)
H25A	1.0439	0.2101	−0.2180	0.161*	0.34 (6)
C26A	0.990 (2)	0.155 (2)	−0.094 (6)	0.125 (15)	0.34 (6)
H26A	1.0300	0.1709	−0.0232	0.150*	0.34 (6)
C27A	0.923 (3)	0.1079 (19)	−0.084 (4)	0.080 (9)	0.34 (6)
H27A	0.9174	0.0922	−0.0058	0.096*	0.34 (6)
C20A	0.811 (3)	−0.051 (3)	0.003 (4)	0.056 (8)	0.34 (6)
H20C	0.7807	−0.0935	0.0184	0.067*	0.34 (6)
H20D	0.8661	−0.0471	0.0667	0.067*	0.34 (6)
C21A	0.798 (4)	0.029 (3)	−0.184 (6)	0.060 (7)	0.34 (6)
H21A	0.7564	0.0262	−0.2650	0.071*	0.34 (6)
C22B	0.8812 (14)	0.0986 (13)	−0.158 (2)	0.076 (5)	0.66 (6)
C23B	0.9134 (19)	0.1135 (13)	−0.264 (3)	0.103 (7)	0.66 (6)
H23B	0.8852	0.0945	−0.3402	0.124*	0.66 (6)
C24B	0.988 (2)	0.1569 (12)	−0.255 (4)	0.132 (11)	0.66 (6)
H24B	1.0091	0.1669	−0.3264	0.158*	0.66 (6)
C25B	1.0296 (15)	0.1853 (13)	−0.141 (4)	0.150 (13)	0.66 (6)
H25B	1.0792	0.2143	−0.1357	0.180*	0.66 (6)
C26B	0.9975 (14)	0.1704 (14)	−0.035 (3)	0.128 (9)	0.66 (6)
H26B	1.0256	0.1894	0.0414	0.154*	0.66 (6)
C27B	0.9233 (15)	0.1271 (14)	−0.043 (2)	0.094 (6)	0.66 (6)
H27B	0.9018	0.1171	0.0276	0.113*	0.66 (6)
C28	0.6744 (6)	0.1067 (4)	−0.1129 (7)	0.0641 (19)	
H28	0.6264	0.0907	−0.0735	0.077*	
C29	0.7082 (7)	0.1766 (5)	−0.0520 (10)	0.093 (3)	
H29A	0.7557	0.1948	−0.0888	0.111*	
H29B	0.7319	0.1695	0.0364	0.111*	
C30	0.6307 (9)	0.2297 (6)	−0.0715 (11)	0.120 (4)	
H30A	0.5858	0.2131	−0.0284	0.144*	
H30B	0.6524	0.2751	−0.0363	0.144*	
C31	0.5898 (10)	0.2386 (6)	−0.2066 (12)	0.123 (4)	
H31A	0.5402	0.2713	−0.2162	0.148*	
H31B	0.6336	0.2587	−0.2485	0.148*	
C32	0.5578 (9)	0.1698 (7)	−0.2659 (12)	0.122 (4)	
H32A	0.5334	0.1770	−0.3541	0.146*	
H32B	0.5106	0.1514	−0.2287	0.146*	
C33	0.6344 (7)	0.1158 (5)	−0.2490 (8)	0.086 (3)	
H33A	0.6120	0.0707	−0.2849	0.103*	
H33B	0.6796	0.1323	−0.2917	0.103*	
Cl1	0.74786 (14)	−0.04867 (12)	0.53404 (17)	0.0666 (5)	
N1	0.7461 (5)	0.0513 (3)	−0.0871 (5)	0.0578 (15)	
O1	0.7073 (4)	0.0135 (3)	0.0899 (4)	0.0620 (13)	
S1A	0.837 (2)	−0.0549 (18)	−0.145 (3)	0.067 (5)	0.34 (6)
S1B	0.861 (2)	−0.0454 (11)	−0.121 (2)	0.083 (4)	0.66 (6)
Sn1	0.72177 (3)	−0.02084 (2)	0.31194 (4)	0.04302 (17)	

### Atomic displacement parameters (Å^2^)

**Table:**

	*U*^11^	*U*^22^	*U*^33^	*U*^12^	*U*^13^	*U*^23^
C1	0.037 (3)	0.044 (3)	0.059 (4)	−0.008 (2)	0.010 (3)	−0.002 (3)
C2	0.056 (5)	0.162 (10)	0.056 (4)	0.029 (6)	0.006 (4)	−0.001 (5)
C3	0.060 (6)	0.167 (12)	0.107 (7)	0.032 (7)	−0.010 (5)	−0.004 (8)
C4	0.045 (5)	0.077 (6)	0.144 (8)	0.018 (4)	0.022 (4)	−0.005 (6)
C5	0.081 (7)	0.103 (8)	0.116 (7)	0.023 (6)	0.042 (5)	−0.008 (6)
C6	0.071 (5)	0.099 (6)	0.064 (5)	0.027 (5)	0.020 (4)	−0.009 (5)
C7	0.062 (5)	0.050 (3)	0.072 (4)	0.005 (3)	0.035 (4)	0.006 (3)
C12	0.162 (11)	0.066 (5)	0.090 (7)	−0.007 (6)	0.011 (6)	−0.019 (5)
C11	0.195 (14)	0.075 (6)	0.141 (10)	−0.037 (7)	0.068 (8)	−0.042 (6)
C10	0.154 (12)	0.054 (5)	0.206 (12)	−0.025 (5)	0.116 (9)	−0.031 (6)
C9	0.132 (11)	0.057 (5)	0.201 (12)	0.025 (6)	0.080 (8)	0.022 (6)
C8	0.090 (7)	0.057 (4)	0.113 (7)	0.020 (4)	0.034 (5)	0.014 (4)
C13	0.042 (3)	0.056 (3)	0.056 (4)	0.005 (3)	0.016 (3)	0.004 (3)
C18	0.061 (5)	0.052 (4)	0.199 (12)	−0.002 (4)	0.028 (7)	0.006 (6)
C17	0.083 (7)	0.074 (6)	0.257 (17)	−0.023 (5)	0.016 (9)	0.024 (9)
C16	0.071 (5)	0.104 (6)	0.173 (12)	−0.034 (5)	0.034 (7)	0.011 (8)
C15	0.054 (5)	0.115 (7)	0.117 (8)	−0.004 (5)	0.037 (5)	0.009 (7)
C14	0.051 (4)	0.078 (5)	0.098 (7)	0.005 (4)	0.019 (4)	0.002 (5)
C19	0.086 (6)	0.055 (4)	0.042 (3)	0.008 (3)	0.025 (3)	0.006 (3)
C20B	0.112 (15)	0.075 (9)	0.016 (6)	0.041 (10)	0.015 (8)	−0.020 (5)
C21B	0.114 (12)	0.046 (9)	0.041 (8)	0.014 (7)	0.038 (8)	−0.006 (6)
C22A	0.09 (2)	0.037 (10)	0.082 (18)	0.008 (12)	0.054 (16)	−0.017 (11)
C23A	0.16 (4)	0.048 (16)	0.12 (2)	−0.01 (2)	0.10 (2)	−0.001 (17)
C24A	0.14 (5)	0.06 (2)	0.21 (4)	0.00 (3)	0.13 (4)	0.02 (3)
C25A	0.12 (3)	0.043 (19)	0.28 (5)	0.00 (2)	0.13 (4)	−0.04 (3)
C26A	0.05 (2)	0.09 (3)	0.23 (4)	0.015 (14)	0.02 (2)	−0.01 (3)
C27A	0.053 (16)	0.050 (17)	0.14 (2)	0.033 (12)	0.017 (17)	−0.008 (19)
C20A	0.060 (18)	0.080 (17)	0.021 (13)	0.016 (13)	−0.003 (12)	0.016 (13)
C21A	0.10 (2)	0.042 (15)	0.050 (17)	0.004 (10)	0.037 (15)	0.011 (14)
C22B	0.079 (11)	0.069 (9)	0.093 (12)	0.024 (9)	0.047 (9)	0.015 (9)
C23B	0.112 (17)	0.082 (14)	0.142 (15)	0.032 (10)	0.087 (14)	0.025 (12)
C24B	0.11 (2)	0.066 (14)	0.25 (3)	0.041 (13)	0.12 (2)	0.047 (17)
C25B	0.081 (15)	0.085 (19)	0.29 (4)	0.027 (10)	0.057 (18)	0.05 (2)
C26B	0.071 (12)	0.079 (14)	0.22 (2)	0.021 (9)	0.012 (14)	0.015 (15)
C27B	0.076 (11)	0.070 (14)	0.136 (14)	0.018 (9)	0.021 (10)	−0.015 (11)
C28	0.084 (6)	0.060 (4)	0.052 (4)	0.015 (4)	0.023 (4)	0.005 (3)
C29	0.099 (7)	0.072 (5)	0.099 (7)	0.022 (5)	0.006 (5)	−0.027 (5)
C30	0.157 (11)	0.084 (7)	0.116 (7)	0.055 (7)	0.021 (7)	−0.013 (6)
C31	0.145 (11)	0.097 (7)	0.126 (8)	0.057 (7)	0.024 (7)	0.017 (7)
C32	0.116 (10)	0.124 (8)	0.108 (8)	0.039 (7)	−0.013 (7)	0.014 (6)
C33	0.106 (8)	0.078 (6)	0.065 (5)	0.011 (5)	0.002 (5)	0.003 (4)
Cl1	0.0649 (12)	0.0897 (14)	0.0434 (9)	−0.0031 (10)	0.0077 (8)	0.0144 (9)
N1	0.080 (4)	0.056 (3)	0.044 (3)	0.015 (3)	0.026 (3)	0.005 (2)
O1	0.082 (4)	0.074 (3)	0.034 (2)	0.011 (3)	0.020 (2)	0.006 (2)
S1A	0.097 (12)	0.051 (7)	0.057 (9)	0.020 (6)	0.030 (7)	0.003 (6)
S1B	0.137 (12)	0.062 (5)	0.064 (6)	0.028 (6)	0.050 (7)	0.001 (4)
Sn1	0.0411 (3)	0.0463 (3)	0.0426 (3)	0.0028 (2)	0.01113 (18)	0.00347 (19)

### Geometric parameters (Å, º)

**Table:**

Sn1—C1	2.141 (4)	C21B—S1B	1.86 (4)
Sn1—C7	2.130 (4)	C22A—C23A	1.3900
Sn1—C13	2.119 (4)	C22A—C27A	1.3900
Sn1—Cl1	2.4439 (19)	C22A—C21A	1.47 (4)
Sn1—O1	2.488 (4)	C23A—H23A	0.9300
C1—C2	1.3900	C23A—C24A	1.3900
C1—C6	1.3900	C24A—H24A	0.9300
C2—H2	0.9300	C24A—C25A	1.3900
C2—C3	1.3900	C25A—H25A	0.9300
C3—H3	0.9300	C25A—C26A	1.3900
C3—C4	1.3900	C26A—H26A	0.9300
C4—H4	0.9300	C26A—C27A	1.3900
C4—C5	1.3900	C27A—H27A	0.9300
C5—H5	0.9300	C20A—H20C	0.9700
C5—C6	1.3900	C20A—H20D	0.9700
C6—H6	0.9300	C20A—S1A	1.76 (5)
C7—C12	1.3900	C21A—H21A	0.9800
C7—C8	1.3900	C21A—N1	1.52 (6)
C12—H12	0.9300	C21A—S1A	1.71 (7)
C12—C11	1.3900	C22B—C23B	1.3900
C11—H11	0.9300	C22B—C27B	1.3900
C11—C10	1.3900	C23B—H23B	0.9300
C10—H10	0.9300	C23B—C24B	1.3900
C10—C9	1.3900	C24B—H24B	0.9300
C9—H9	0.9300	C24B—C25B	1.3900
C9—C8	1.3900	C25B—H25B	0.9300
C8—H8	0.9300	C25B—C26B	1.3900
C13—C18	1.3900	C26B—H26B	0.9300
C13—C14	1.3900	C26B—C27B	1.3900
C18—H18	0.9300	C27B—H27B	0.9300
C18—C17	1.3900	C28—H28	0.9800
C17—H17	0.9300	C28—C29	1.519 (12)
C17—C16	1.3900	C28—C33	1.499 (11)
C16—H16	0.9300	C28—N1	1.501 (10)
C16—C15	1.3900	C29—H29A	0.9700
C15—H15	0.9300	C29—H29B	0.9700
C15—C14	1.3900	C29—C30	1.536 (13)
C14—H14	0.9300	C30—H30A	0.9700
C19—C20B	1.56 (3)	C30—H30B	0.9700
C19—C20A	1.44 (4)	C30—C31	1.491 (15)
C19—N1	1.317 (9)	C31—H31A	0.9700
C19—O1	1.257 (8)	C31—H31B	0.9700
C20B—H20A	0.9700	C31—C32	1.488 (17)
C20B—H20B	0.9700	C32—H32A	0.9700
C20B—S1B	1.76 (2)	C32—H32B	0.9700
C21B—H21B	0.9800	C32—C33	1.537 (14)
C21B—C22B	1.50 (2)	C33—H33A	0.9700
C21B—N1	1.46 (3)	C33—H33B	0.9700
			
C2—C1—C6	120.0	C26A—C27A—C22A	120.0
C2—C1—Sn1	121.3 (3)	C26A—C27A—H27A	120.0
C6—C1—Sn1	118.7 (3)	C19—C20A—H20C	109.5
C1—C2—H2	120.0	C19—C20A—H20D	109.5
C1—C2—C3	120.0	C19—C20A—S1A	111 (2)
C3—C2—H2	120.0	H20C—C20A—H20D	108.1
C2—C3—H3	120.0	S1A—C20A—H20C	109.5
C2—C3—C4	120.0	S1A—C20A—H20D	109.5
C4—C3—H3	120.0	C22A—C21A—H21A	108.1
C3—C4—H4	120.0	C22A—C21A—N1	108 (4)
C5—C4—C3	120.0	C22A—C21A—S1A	118 (4)
C5—C4—H4	120.0	N1—C21A—H21A	108.1
C4—C5—H5	120.0	N1—C21A—S1A	107 (3)
C6—C5—C4	120.0	S1A—C21A—H21A	108.1
C6—C5—H5	120.0	C23B—C22B—C21B	118.2 (12)
C1—C6—H6	120.0	C23B—C22B—C27B	120.0
C5—C6—C1	120.0	C27B—C22B—C21B	121.1 (12)
C5—C6—H6	120.0	C22B—C23B—H23B	120.0
C12—C7—C8	120.0	C22B—C23B—C24B	120.0
C12—C7—Sn1	120.1 (3)	C24B—C23B—H23B	120.0
C8—C7—Sn1	119.9 (3)	C23B—C24B—H24B	120.0
C7—C12—H12	120.0	C23B—C24B—C25B	120.0
C11—C12—C7	120.0	C25B—C24B—H24B	120.0
C11—C12—H12	120.0	C24B—C25B—H25B	120.0
C12—C11—H11	120.0	C26B—C25B—C24B	120.0
C12—C11—C10	120.0	C26B—C25B—H25B	120.0
C10—C11—H11	120.0	C25B—C26B—H26B	120.0
C11—C10—H10	120.0	C25B—C26B—C27B	120.0
C9—C10—C11	120.0	C27B—C26B—H26B	120.0
C9—C10—H10	120.0	C22B—C27B—H27B	120.0
C10—C9—H9	120.0	C26B—C27B—C22B	120.0
C10—C9—C8	120.0	C26B—C27B—H27B	120.0
C8—C9—H9	120.0	C29—C28—H28	107.0
C7—C8—H8	120.0	C33—C28—H28	107.0
C9—C8—C7	120.0	C33—C28—C29	111.6 (7)
C9—C8—H8	120.0	C33—C28—N1	113.1 (6)
C18—C13—C14	120.0	N1—C28—H28	107.0
C18—C13—Sn1	118.4 (3)	N1—C28—C29	110.8 (7)
C14—C13—Sn1	121.5 (3)	C28—C29—H29A	109.9
C13—C18—H18	120.0	C28—C29—H29B	109.9
C13—C18—C17	120.0	C28—C29—C30	109.0 (9)
C17—C18—H18	120.0	H29A—C29—H29B	108.3
C18—C17—H17	120.0	C30—C29—H29A	109.9
C16—C17—C18	120.0	C30—C29—H29B	109.9
C16—C17—H17	120.0	C29—C30—H30A	109.4
C17—C16—H16	120.0	C29—C30—H30B	109.4
C17—C16—C15	120.0	H30A—C30—H30B	108.0
C15—C16—H16	120.0	C31—C30—C29	111.0 (9)
C16—C15—H15	120.0	C31—C30—H30A	109.4
C14—C15—C16	120.0	C31—C30—H30B	109.4
C14—C15—H15	120.0	C30—C31—H31A	109.3
C13—C14—H14	120.0	C30—C31—H31B	109.3
C15—C14—C13	120.0	H31A—C31—H31B	108.0
C15—C14—H14	120.0	C32—C31—C30	111.5 (10)
C20A—C19—C20B	19 (2)	C32—C31—H31A	109.3
N1—C19—C20B	113.4 (10)	C32—C31—H31B	109.3
N1—C19—C20A	112.3 (16)	C31—C32—H32A	109.5
O1—C19—C20B	122.6 (10)	C31—C32—H32B	109.5
O1—C19—C20A	122.1 (17)	C31—C32—C33	110.7 (10)
O1—C19—N1	123.8 (7)	H32A—C32—H32B	108.1
C19—C20B—H20A	111.0	C33—C32—H32A	109.5
C19—C20B—H20B	111.0	C33—C32—H32B	109.5
C19—C20B—S1B	104.0 (13)	C28—C33—C32	109.5 (8)
H20A—C20B—H20B	109.0	C28—C33—H33A	109.8
S1B—C20B—H20A	111.0	C28—C33—H33B	109.8
S1B—C20B—H20B	111.0	C32—C33—H33A	109.8
C22B—C21B—H21B	108.2	C32—C33—H33B	109.8
C22B—C21B—S1B	111 (2)	H33A—C33—H33B	108.2
N1—C21B—H21B	108.2	C19—N1—C21B	117.1 (14)
N1—C21B—C22B	117.3 (17)	C19—N1—C21A	115 (2)
N1—C21B—S1B	103.6 (16)	C19—N1—C28	120.9 (6)
S1B—C21B—H21B	108.2	C21B—N1—C21A	14 (3)
C23A—C22A—C27A	120.0	C21B—N1—C28	121.9 (13)
C23A—C22A—C21A	118 (2)	C28—N1—C21A	123 (2)
C27A—C22A—C21A	121 (2)	C19—O1—Sn1	135.9 (5)
C22A—C23A—H23A	120.0	C21A—S1A—C20A	93 (3)
C24A—C23A—C22A	120.0	C20B—S1B—C21B	92.3 (14)
C24A—C23A—H23A	120.0	C1—Sn1—Cl1	98.31 (14)
C23A—C24A—H24A	120.0	C1—Sn1—O1	84.26 (18)
C25A—C24A—C23A	120.0	C7—Sn1—C1	118.5 (2)
C25A—C24A—H24A	120.0	C7—Sn1—Cl1	95.29 (16)
C24A—C25A—H25A	120.0	C7—Sn1—O1	87.11 (19)
C24A—C25A—C26A	120.0	C13—Sn1—C1	118.0 (2)
C26A—C25A—H25A	120.0	C13—Sn1—C7	120.2 (2)
C25A—C26A—H26A	120.0	C13—Sn1—Cl1	94.63 (17)
C25A—C26A—C27A	120.0	C13—Sn1—O1	80.4 (2)
C27A—C26A—H26A	120.0	Cl1—Sn1—O1	175.07 (14)
C22A—C27A—H27A	120.0		
			
C1—C2—C3—C4	0.0	C23A—C24A—C25A—C26A	0.0
C2—C1—C6—C5	0.0	C24A—C25A—C26A—C27A	0.0
C2—C1—Sn1—C7	−61.0 (4)	C25A—C26A—C27A—C22A	0.0
C2—C1—Sn1—C13	98.6 (4)	C27A—C22A—C23A—C24A	0.0
C2—C1—Sn1—Cl1	−161.6 (3)	C27A—C22A—C21A—N1	53 (5)
C2—C1—Sn1—O1	22.6 (4)	C27A—C22A—C21A—S1A	−68 (5)
C2—C3—C4—C5	0.0	C20A—C19—C20B—S1B	68 (6)
C3—C4—C5—C6	0.0	C20A—C19—N1—C21B	−18 (2)
C4—C5—C6—C1	0.0	C20A—C19—N1—C21A	−3 (3)
C6—C1—C2—C3	0.0	C20A—C19—N1—C28	165 (2)
C6—C1—Sn1—C7	117.3 (3)	C20A—C19—O1—Sn1	39 (3)
C6—C1—Sn1—C13	−83.2 (4)	C21A—C22A—C23A—C24A	−173 (4)
C6—C1—Sn1—Cl1	16.6 (3)	C21A—C22A—C27A—C26A	173 (4)
C6—C1—Sn1—O1	−159.1 (4)	C22B—C21B—N1—C19	−105 (2)
C7—C12—C11—C10	0.0	C22B—C21B—N1—C21A	169 (17)
C12—C7—C8—C9	0.0	C22B—C21B—N1—C28	72 (3)
C12—C7—Sn1—C1	46.3 (4)	C22B—C21B—S1B—C20B	100.0 (17)
C12—C7—Sn1—C13	−112.8 (4)	C22B—C23B—C24B—C25B	0.0
C12—C7—Sn1—Cl1	148.7 (3)	C23B—C22B—C27B—C26B	0.0
C12—C7—Sn1—O1	−35.6 (4)	C23B—C24B—C25B—C26B	0.0
C12—C11—C10—C9	0.0	C24B—C25B—C26B—C27B	0.0
C11—C10—C9—C8	0.0	C25B—C26B—C27B—C22B	0.0
C10—C9—C8—C7	0.0	C27B—C22B—C23B—C24B	0.0
C8—C7—C12—C11	0.0	C28—C29—C30—C31	−56.4 (14)
C8—C7—Sn1—C1	−134.1 (4)	C29—C28—C33—C32	−58.0 (12)
C8—C7—Sn1—C13	66.9 (4)	C29—C28—N1—C19	90.0 (10)
C8—C7—Sn1—Cl1	−31.6 (4)	C29—C28—N1—C21B	−87.1 (16)
C8—C7—Sn1—O1	144.1 (4)	C29—C28—N1—C21A	−103 (3)
C13—C18—C17—C16	0.0	C29—C30—C31—C32	57.4 (16)
C18—C13—C14—C15	0.0	C30—C31—C32—C33	−57.3 (16)
C18—C13—Sn1—C1	−7.3 (4)	C31—C32—C33—C28	57.0 (14)
C18—C13—Sn1—C7	151.8 (4)	C33—C28—C29—C30	57.6 (12)
C18—C13—Sn1—Cl1	−109.3 (4)	C33—C28—N1—C19	−143.8 (8)
C18—C13—Sn1—O1	70.9 (4)	C33—C28—N1—C21B	39.1 (17)
C18—C17—C16—C15	0.0	C33—C28—N1—C21A	23 (3)
C17—C16—C15—C14	0.0	N1—C19—C20B—S1B	−22.9 (17)
C16—C15—C14—C13	0.0	N1—C19—C20A—S1A	−7 (3)
C14—C13—C18—C17	0.0	N1—C19—O1—Sn1	−157.1 (6)
C14—C13—Sn1—C1	176.1 (3)	N1—C21B—C22B—C23B	−148.8 (19)
C14—C13—Sn1—C7	−24.8 (4)	N1—C21B—C22B—C27B	41 (3)
C14—C13—Sn1—Cl1	74.1 (4)	N1—C21B—S1B—C20B	−26.7 (18)
C14—C13—Sn1—O1	−105.7 (4)	N1—C21A—S1A—C20A	−13 (4)
C19—C20B—S1B—C21B	27.5 (17)	N1—C28—C29—C30	−175.3 (8)
C19—C20A—S1A—C21A	12 (4)	N1—C28—C33—C32	176.3 (9)
C19—O1—Sn1—C1	175.4 (7)	O1—C19—C20B—S1B	162.3 (11)
C19—O1—Sn1—C7	−65.6 (7)	O1—C19—C20A—S1A	158.2 (17)
C19—O1—Sn1—C13	55.7 (7)	O1—C19—N1—C21B	177.3 (15)
C19—O1—Sn1—Cl1	53.7 (18)	O1—C19—N1—C21A	−168 (3)
C20B—C19—C20A—S1A	−104 (8)	O1—C19—N1—C28	0.1 (12)
C20B—C19—N1—C21B	2.6 (17)	S1A—C21A—N1—C19	11 (4)
C20B—C19—N1—C21A	18 (3)	S1A—C21A—N1—C21B	112 (17)
C20B—C19—N1—C28	−174.6 (13)	S1A—C21A—N1—C28	−156 (2)
C20B—C19—O1—Sn1	17.1 (17)	S1B—C21B—C22B—C23B	92.4 (18)
C21B—C22B—C23B—C24B	−170 (2)	S1B—C21B—C22B—C27B	−78 (2)
C21B—C22B—C27B—C26B	170 (2)	S1B—C21B—N1—C19	17.8 (19)
C22A—C23A—C24A—C25A	0.0	S1B—C21B—N1—C21A	−68 (14)
C22A—C21A—N1—C19	−116 (3)	S1B—C21B—N1—C28	−165.0 (11)
C22A—C21A—N1—C21B	−15 (12)	Sn1—C1—C2—C3	178.2 (4)
C22A—C21A—N1—C28	77 (4)	Sn1—C1—C6—C5	−178.3 (4)
C22A—C21A—S1A—C20A	109 (4)	Sn1—C7—C12—C11	179.7 (4)
C23A—C22A—C27A—C26A	0.0	Sn1—C7—C8—C9	−179.7 (4)
C23A—C22A—C21A—N1	−134 (3)	Sn1—C13—C18—C17	−176.6 (4)
C23A—C22A—C21A—S1A	106 (4)	Sn1—C13—C14—C15	176.5 (4)
